# Taxation of tobacco, alcohol, and sugar-sweetened beverages: reviewing the evidence and dispelling the myths

**DOI:** 10.1136/bmjgh-2023-011866

**Published:** 2023-10-09

**Authors:** Guillermo R Paraje, Prabhat Jha, William Savedoff, Alan Fuchs

**Affiliations:** 1 Escuela de Negocios, Universidad Adolfo Ibáñez, Santiago de Chile, Chile; 2 Millennium Nucleus for the Evaluation and Analysis of Drug Policies (nDP), Santiago de Chile, Chile; 3 CGHR, Centre for Global Health Research, St. Michael’s Hospital and Dalla Lana School of Public Health, University of Toronto, Toronto, Ontario, Canada; 4 Social Insight, Washington, Maine, USA; 5 World Bank, Washington, District of Columbia, USA

**Keywords:** Health economics, Public Health, Review

## Abstract

The article reviews the large body of evidence on how taxation affects the consumption of tobacco, alcohol, and sugar-sweetened beverages (SSB). There is abundant evidence that demand for tobacco, alcohol, and SSB is price-responsive and that tax changes are quickly passed on to consumers. This suggests that taxes can be highly effective in changing consumption and reducing the burden of diseases associated with consuming these products. Tobacco, alcohol, and SSB industries oppose taxation on similar grounds, mostly on the regressivity of taxes since regressive taxes take a larger percentage of income from low income earners than from middle and high income earners; but also on the effects taxes might have on employment and economic activity; and, in the case of tobacco, the effects taxation has on illicit trade.

Contrary to industry arguments, evidence shows that taxation may have short-term negative financial consequences for low-income households. However, medium and long-term financial benefits from reduced healthcare costs, better health, and welfare largely compensate for such consequences. Moreover, taxation does not negatively affect aggregate economic activity or employment, as consumers switch demand to other products that generate employment and may compensate for any employment loss in taxed sectors. Evidence also shows the revenues generated are generally spent on labour-intensive services. In the case of illicit trade in tobacco, evidence shows that illicit trade has not increased globally (rather the opposite) despite increases in tobacco taxes. Profit-maximising smugglers increase illicit cigarette prices along with the increases in licit cigarette prices. This implies that even when increased taxes divert some demand to the illicit market, they push prices up in the illicit market, discouraging consumption.

**Summary box**

- Health taxes (on tobacco, alcohol, and SSB) are effective economic instruments to change people’s behaviour and reduce the consumption of unhealthy products.
- The article summarises and critically discusses the large body of evidence regarding the effect of taxes on reducing the consumption of tobacco, alcohol, and SSB.
- The article also provides consolidated evidence regarding the lack of economic substance behind common arguments that industries use to oppose these taxes (eg, illicit trade, negative effects on employment, regressivity).
- Evidence provided in this article can be used to design and implement health taxes more effectively.

## Introduction

Even as humans live longer and generally healthier lives, consuming tobacco, alcohol, and processed foods with sugars is causing substantial death and disease. In recent decades, the magnitude of these health effects has become increasingly apparent in terms of cancers, cardiovascular disorders, diabetes, and other illnesses that hinder healthy ageing and social development. Fortunately, strong evidence is available to demonstrate the efficacy of public health policies that can reduce consumption, foremost among which are health taxes.

It is estimated that every year tobacco kills between 5 and 8 million people worldwide (mostly in low- and middle-income countries), and a more uncertain but high number of secondhand (passive) smokers.^1 2^ Although tobacco consumption is still high, it is falling, representing a reversal of the rising trends before the late 1990s.^3^ It is estimated that one smoking death occurs for every 0.8 to 1.1 million cigarettes smoked.^4^ Thus, the 7.4 trillion cigarettes consumed in 2019 alone will eventually lead to at least 7 million premature deaths.

In the case of alcohol, although the prevalence of current drinkers aged 15 years and older decreased between 2000 and 2016 (from 47.6% to 43%), the per capita volume of pure alcohol consumed has increased from 5.7 litres to 6.4 litres and is projected to further grow to 7 litres by 2025.^5 6^ If only drinkers are considered, pure alcohol consumption per capita reached 15.1 litres in 2016 (up from 11.1 litres in 2000).^5^ Furthermore, considering global population growth in 2000–2016, the number of drinkers worldwide increased by 16%, and the amount of pure alcohol consumed globally increased by 58% (an annual average increase of 3.1% vs a 1.7% average annual increase in global constant per capita gross domestic product (GDP)).^7 8^

Consumption of sugar-sweetened beverages (SSB) has been increasing although there are few estimates of the evolution of consumption globally. Global consumption for carbonated soft drinks (excluding bottled still or carbonated water, fruit or vegetable juices, coffee, tea, or sports drinks) increased from 36 litres per person per year in 1997 to 43.1 litres in 2010.^9^ Recent estimates have shown that consumption of SSB was projected to stabilise (or decrease slightly) by 2022 at a very high level of consumption for high-income countries (at about 120 litres per year per capita) and for upper-middle-income countries (at about 50 litres per year per capita), but increase strongly for lower-middle and low-income countries.^10^

Naturally, consuming products with significant and negative impacts on health has economic implications. Such consequences can be broadly separated into two groups of costs: i) direct costs attributable to the treatment of diseases; and ii) indirect costs that can be attributed to premature mortality, loss of productivity due to absenteeism and presentism (ie, people going to their jobs but being less productive because of disabilities, illnesses, etc.), opportunity costs of caregivers, suffering and pain, etc. It has been estimated that the economic cost of tobacco consumption at the global level was equivalent to 1.8% of the world GDP in 2012 (about 1.8 trillion in US dollar purchasing power parities (USD PPP).^11^ Of that amount, direct costs (related to healthcare expenditures) totaled USD PPP 467 billion (equivalent to 5.7% of global health expenditures). Pooled results for 29 locations (countries, states/regions, cities) found that the total cost of alcohol consumption amounted to USD PPP 817 per adult, equivalent to 1.5% of the GDP (of which 68% were indirect costs).^12^

Fiscal tools, such as taxes, can play a fundamental role in preventing chronic diseases and improving people’s lives and social outcomes. This article presents current evidence on taxation as a proven tool to discourage the consumption of harmful products (tobacco, alcohol, and SSB) and thereby improve population health. It discusses the economic rationale for taxing these products and documents the health effects and costs to society. It presents evidence on the effectiveness of taxation in raising prices and reducing consumption, along with showing how considering health benefits and indirect financial effects, taxation disproportionately benefits poorer households. It also considers the role of industries in opposing taxation. The article differs from previous reviews in its scope (covering all three products) and in its comprehensiveness (in addressing the arguments for and against health taxes, including those arguments used by the industry).

For this article, tobacco products are defined as those containing tobacco that can be smoked, inhaled, chewed, etc. Alcoholic beverages addressed here are beer, wine, and liquor. SSB refers to processed drinks with added sugars. Health taxes are relevant to other products which are, however, beyond the scope of this article and raise additional issues. These other products include so-called “electronic cigarettes” (electronic nicotine delivery systems or electronic non-nicotine delivery systems); beverages with added alcohol (eg, fruit flavoured seltzer); and fruit juices and ultra-processed food. Some countries have indeed implemented health taxes on some of these products (Mexico, for instance, implemented a tax on non-essential food with high energy density^13^) but they are less well researched.

## Effectiveness of tax policies to decrease consumption

The consumption of tobacco, alcohol, and SSB is associated with negative externalities, as such, consumption negatively affects the well-being of third parties (externalities occur when one individual’s action – consumption, production – affects the well-being of another individual). Not only negative externalities are present in the consumption of these products, but also negative “internalities”, which arise from individuals ignoring or not correctly considering harmful health effects to themselves.^14^ For instance, tobacco externalities may arise from the health effect on passive smokers,^1 2^ while alcohol externalities involve traffic road accidents, domestic and street violence, etc.^15^ In the case of SSB, as in the case of tobacco and alcohol, externalities arise from the healthcare costs related to the several conditions associated with SSB consumption.^16^ In all cases, family suffering associated with pain and illnesses can also be considered externalities.

The usual tool used to correct negative externalities are the so-called Pigouvian taxes that increase the prices consumers pay by enough to offset the costs of present and future externalities. Studies have consistently shown that taxes on tobacco, alcohol, and SSB effectively raise prices and reduce consumption. This effectiveness results from how demand responds to product price increases (own-price elasticity).

### Pass-through of taxes to prices

Although it is common to assume that a tax increase automatically raises prices, the degree and speed to which prices rise will depend, among other things, on the market structure, the structure of the excise tax system, and the availability of non-taxed or differentially taxed substitutes (which affects the own-price demand elasticity). In particular, prices will typically rise more in response to a tax increase when the market is concentrated (as they often are in the case of these products), the taxes are charged at the retail level, and when a product has few or no close substitutes (and, hence, a low own-price demand elasticity). In addition, taxes can be fully passed to prices (pass-through equal to 1), can be undershifted (pass-through less than 1), or can be overshifted (pass-through greater than 1). Evidence shows a relationship between market power and the firm’s ability to pass entirely or even overshift taxes to prices, which has been found for many industries.^17 18^

For example, in the US, the 1983 increase in the Federal Cigarette Excise Tax was overshifted by oligopolistic firms exploiting their market power.^19^ Similar behaviour was found in Europe.^17^ In the European case, ad-valorem taxes (ie, a percentage of the price) tend to produce lower pass-through rates for tobacco when compared with specific taxes (ie, a tax per unit sold). Tax structure can also affect pass-through rates. In India^20^ and Bangladesh,^21^ a complex tiered tax system has given the tobacco industry room for passing taxes differentially according to price segments (ie, taxes are undershifted for cheaper cigarettes and overshifted for premium cigarettes, thereby leading to downward substitution rather than reducing consumption). The perspective of further regulations may also affect the pass-through rate, as producers facing a scenario of tightening restrictions in consumption, higher taxation, etc., may choose to maximise short-run profits by overshifting taxes.^22^ There is evidence that the tobacco industry uses several strategies (eg, stockpiling – oversupplying the domestic market before a tax increase) to delay the price change after a tax increase, although, eventually, prices increase as a result of such an increase.^23 24^

For alcohol, taxes are also fully passed or overshifted. A review found that taxes are generally overshifted in the case of beer and fully shifted for wine and spirits.^25^ In the US, state and federal alcohol taxes appear to be overshifted, especially for beer and spirits. The price adjustment is also quite rapid, within 3 months of the tax change.^26^ For the UK, the pass-through rate varies by the price level of products, as producers of relatively cheaper alcoholic beverages tend to undershift taxes. In contrast, those of relatively more expensive beverages overshift them.^27^ A similar finding was reported for the pass-through of alcohol taxes for 27 Organization for Economic Cooperation and Development (OECD) countries.^28^

In the case of SSB, the evidence is mixed. A recent meta-analysis found that, on average, the pass-through was 70% of the tax, although with considerable variation across studies.^29^ In Mexico, the SSB tax was mainly overshifted for sodas and fully or mildly undershifted for other SSBs.^30 31^ In France, taxes were overshifted for sodas and undershifted for fruit juices and flavoured waters.^32^ In Denmark, three tax changes between 1998 and 2003 were either fully shifted or overshifted.^33^ However, in other cases, taxes have been undershifted. This is the case of Barbados,^34^ Chile^35^ and some categories of SSB in the UK (eg, those in the top tier of the tax),^36^ etc. In the US, studies of local taxes have found mixed results, depending on the location of the store (proximity to state borders led to undershifting),^37^ store, and beverage types.^38 39^

### Price responsiveness of demand

Studies have measured the own-price elasticities of tobacco and have centred around values of −0.4 (ie, a 10% increase in prices implies a 4% decrease in quantity consumed) for high-income countries and around −0.5 for low- and middle-income countries, although the difference may not be statistically significant.^40–43^ About half of the own-price elasticity is due to a decrease in prevalence (ie, due to people quitting smoking), while the other half is due to a reduction in the number of cigarettes smoked by those who continue smoking.^40 44^ Youths are substantially more price-responsive than adults in countries at all income levels,^40 45 46^ and young men are more price-responsive than young women.^47^ In addition, higher prices delayed or prevented smoking initiation in various countries (ie, the own-price elasticity of smoking onset is negative and statistically significant).^48–52^ Finally, higher tobacco prices increase the likelihood of cessation in both high- and middle-income countries.^53–58^

The evidence on own-price elasticity for alcohol is also compelling, with values around −0.5.^59^ However, not all types of alcoholic beverages are equal, as beer demand is less price-responsive than wine and spirits (-0.3 vs −0.6, respectively).^41 60^ There is little evidence of gender differences in price responsiveness.^59^ Studies show different results on own-price elasticities by age, finding no conclusive differences between youths and adults.^61 62^ Even binge drinkers are price-responsive^63^ but tend to choose cheaper drinks to keep up their alcohol consumption. This implies that policies aiming at increasing the price of more affordable drinks (eg, minimum unit price policies) can effectively reduce binge and/or heavy drinking.^61 64 65^ Finally, evidence on how prices affect alcohol initiation is scarce but suggests that higher prices delay and, to some extent, prevent initiation. This can have long-lasting effects on future drinking patterns; for example, individuals who initiate at older ages have a lower probability of having frequent heavy-drinking episodes.^66 67^

For SSB, there is mounting evidence of relatively high (in absolute terms) and significantly negative own-price elasticities. The own-price elasticity for SSBs is around −1 (eg, a 10% increase in SSB prices decreases consumption by 10%).^68^ A systematic review of studies in 164 countries found that price-responsiveness was higher in the lowest-income countries and for the youngest and oldest adults (vis-à-vis middle-aged ones, probably reflecting life-cycle changes in incomes). However, it found no differences in price-responsiveness between men and women.^69^

## Challenges from the industry

The tobacco industry has a long and well-researched history of concealing evidence of the toxicity of its products, deceiving the public about the harmful effects of tobacco consumption, and interfering in public policy. As early as the 1950s, the tobacco companies concealed evidence of their products' harmfulness and nicotine’s addictiveness. In the 1970s and the 1980s, they denied links between smoking and cancers and the harmfulness of secondhand smoking.^70 71^ More recently, despite robust scientific evidence, they misled the public regarding the relative safety of “light” or “low-tar” cigarettes.^70^ The tobacco industry’s tactics have been exposed through successful efforts to make the industry’s documents public.^72^ These documents have demonstrated the large discrepancies between what the industry knew and the ideas it promoted.

Apart from concealing and distorting evidence, the tobacco industry has given several arguments to deter, impede or delay increases in tobacco taxes. A systematic review of the tobacco industry’s tobacco tax opposition found that the most common and consistent arguments were that tobacco taxes (a) are regressive; (b) lead to more illicit trade and foster organised crime; and (c) reduce employment and harm businesses.^73^ The tactics of the alcohol and SSB (food, in general) industries to oppose taxes and regulations are remarkably similar to those used by the tobacco industry. They also shift blame for unhealthy eating or drinking away from themselves as purveyors of products and onto the individuals who are intentionally influenced by marketing campaigns.^71^ The alcohol and processed food industries have also linked regulations and taxes to the loss of personal freedoms and the notion that public health policies create “food police” or “food fascism”.^71 74^ Self-regulation is often proposed as an effective and more efficient alternative to state regulations even when this has been shown to be ineffective. Like the tobacco industry, the producers of alcohol and SSB lobby governments and the public by arguing that taxes do not reduce consumption; that they are regressive; and that they are “discriminatory” (as they are levied on specific groups of products) or even unconstitutional.^75^

### Regressivity of taxes

Common arguments from tobacco, alcohol, and SSB industries claim that because poorer individuals spend more on these products as a proportion of their budget, any price increase induced by tax changes will affect them disproportionally more than, for instance, richer individuals. Hence, these taxes are regressive. However, tobacco, alcohol, and SSB are unlike other taxable goods. Preventing health and economic harms associated with consuming these products generate large benefits (in health improvements, healthcare cost reductions, and higher disposable income for purchasing non-toxic goods and services) to current and potential consumers. Moreover, the financial impact of these prevented costs is disproportionally higher for poorer households, and they more than offset any negative immediate financial costs that taxation may have on them. Several studies have been conducted using the extended cost-benefit analysis (ECBA) methodology to test this hypothesis. ECBA incorporates any short-term welfare losses from excise taxes into a framework that includes medium- and long-term health benefits for those who quit or consume fewer harmful products. Among other aspects, it accounts for differential behavioural responses across population groups—including different income groups—by estimating specific group price elasticities.

The ECBA methodology has been applied widely, including in a 13-country study.^76^ At least 13 country studies where the ECBA has been implemented highlight that the medium- and long-term benefits of reducing smoking can outweigh the short-term tax spending, resulting in net gains, particularly among poorer households.^77–86^ Similar findings are reached by Fuchs, Paz and Gonzalez Icaza, who simulate tax policy changes in eight low-, middle- and high-income countries.^87^ The distribution of elasticities and resultant health and economic benefits from reduced medical expenses and lower years of working-life lost (YWLL) generally more than offset the short-term negative effects of tobacco taxes on household budgets. The tax incidence is progressive for sufficiently high price shocks and elasticity scenarios. Half the population in these countries could benefit from net positive income gains in the medium- to long-term if cigarette prices rose by 50 percent. In a similar study, Postolovska *et al* find that increasing the cigarette excise tax rate to 75 percent of the retail price could raise large health and financial benefits to Armenian households, with pro-poor impacts.^88^

The reduction in medical bills is the most significant component driving the net benefits under the ECBA. All income groups benefit from reduced medical expenses when taxes discourage smoking, but these benefits are disproportionately larger for poor households. In Moldova, where tobacco-related diseases are the leading cause of premature adult deaths, just accounting for reductions in medical expenses is enough to offset the initial negative price effect on household expenditures with a clear progressive pattern.^79^ In Chile, Ukraine, and Russia, reducing medical expenses constitutes the largest long-term benefit of the tobacco price increase under the ECBA.^78 80 81^ Similarly, tobacco price increases have positive welfare gains from being able to work longer. In Bangladesh, for instance, the main gains of taxing tobacco under the ECBA model arise from extending peoples’ working lives (ie, lowering YWLLs).^77^

Complete studies on the distributional consequences of taxing SSB are less common but growing in number. For example, a study of excise taxes on SSB in Ukraine found that the net effect is progressive, although small in magnitude: raising SSB prices by 20 percent increases the disposable income of the poorest quintile of the population by 0.03 percent.^89^ In Kazakhstan, where the average price elasticity of SSB is estimated to be −0.70, lower-income deciles benefit more than higher-income deciles from a simulated introduction of a 20 percent price increase of SSB.^85^ Finally, a recent ECBA in Brazil shows that price increases on alcohol, tobacco, and SSB have positive and progressive effects when incorporating the impact of health taxes on prices, medical expenditures, and productive lives.^90^ A recent report analysed the distributional effect of alcohol taxes in the UK, finding no evidence to support the idea that alcohol taxes are regressive.^91^ Furthermore, once the use of alcohol tax revenues by the Government is considered, results can be strongly progressive if revenues are used to finance increased health or other pro-poor programmes. Note that “soft” earmarking (ie, that which is not legally required) helps overcome political opposition to higher tobacco excise taxes.^24^ The same effect can be found for SSB taxes: in the case of the Philadelphia SSB tax, for instance, a significant proportion of revenues were allocated to fund preschool education and community schools.^92^

In the case of alcohol, the relative financial burden may be affected not only by income but also, crucially, by the intensity of drinking. In Australia, alcohol taxes represent a higher burden to heavy drinkers, irrespective of their incomes. Because of that, Minimum Unit Pricing (MUP) policies or taxes that increase the cost of the cheapest alcohol can be more effective in reducing alcohol consumption without having highly regressive effects.^93^ However, this partial study does not consider the distributional impact on revenues and healthcare cost savings.

### Taxes and illicit trade

One of the common arguments the tobacco and alcohol industries have used to challenge health taxes is to claim that these policies will be ineffective because they will encourage illicit trade. While illicit trade can undercut prices in the market, there is little evidence that it has undermined the effectiveness of taxes in raising overall prices. Furthermore, price differences are not a significant factor in explaining illicit trade; other factors such as tax administration, enforcement and political governance are of much greater significance. Finally, the kinds of large-scale illicit trade that could affect markets are unlikely without direct or indirect complicity by the companies that manufacture products in these highly concentrated markets. Indeed, investigations have found that the transnational tobacco industry is responsible for producing about two-thirds of illicitly traded cigarettes.^94^

While data on illicit trade is difficult to obtain, researchers and investigators have developed sophisticated methods for estimating its scale and sources.^40^ For example, researchers have estimated illicit trade by measuring the gap between registered exports and imports or between household consumption and legal supplies of products. Researchers have studied tobacco smuggling by randomly purchasing cigarettes from smokers or by gathering discarded packets to generate representative samples from which to extrapolate the share and sources of illicit cigarettes. Criminal investigations have documented supply chains for illicit products of all kinds. The information discussed here relies on a combination of such evidence.

Evidence against the tobacco industry’s claims on illicit trade is substantial. First, illicit trade has stayed the same in the last decade despite increasing tobacco taxes.^95–97^ In fact, illicit tobacco products, mostly cigarettes, constitute a stable share in a shrinking market. Cigarettes have become less affordable (measuring affordability as the percentage of per capita GDP needed to purchase 100 packs of cigarettes) in 117 out of 168 countries between 2008 and 2018,^97^ and the lesser affordability came mostly from increasing tobacco taxes. Based on WHO data, the unweighted average of the total tax share (as a proportion of final retail price) for the most sold brand of cigarettes went from 46.6% in 2008 to 52.4% in 2018.^98^ Out of 174 countries with complete data in this period, 118 increased the total tax share, 48 increased it by more than 10 percentage points, and 22 increased it by more than 20 percentage points. There is no significant difference between the richest 40 and the poorest 40 countries, although the first had an initially higher total tax burden (66.5% vs 37.6% in 2008).^98^ Out of 179 countries, 91 increased the tax burden of specific taxes, 58 did it by more than 10 percentage points and 33 by more than 20. A recent exercise in scoring changes in tobacco taxes found that out of 170 countries, 51 increased tobacco taxes between 2014 and 2018.^99^ The global situation of raising taxes and decreasing consumption does not fit the tobacco industry’s narrative nor the evidence on illicit trade.

Second, the evidence cited by tobacco companies to link tobacco tax increases with illicit trade is weak and generally based on studies financed by the tobacco industry. These studies rarely make their methods and data publicly available for peer review and scrutiny. By contrast, independent studies have used cross-country evidence to show that countries with higher taxes have a lower penetration of illicit trade than those with lower taxes.^40 100^ Recent examples are cases in the UK, the Philippines, and Botswana.^101–103^ It is more likely that illicit trade is driven less by tobacco price increases than by tax administration authorities’ general capacity to enforce taxation – and this is true not only for tobacco.^40^ Lack of controls, corruption, and weak administrative capacities may foster illicit trade, although countries with middling administrative capacities, such as the Philippines and Botswana (among others) have succeeded in restraining illicit trade. Strengthening excise tax laws (eg, harsher penalties, strong governance, control processes, etc.) helps curb illicit tobacco trade (as illicit trade in any other product).

Furthermore, smuggling on a scale that could affect prices requires active involvement of industry – as evidenced by guilty pleas in Canada in 2008^104^ – or tacit involvement – as evidenced by exports far exceeding potential domestic demand in countries known to be sources of illicit trade.^105^

For example, from the 1990s until they were found guilty in 2004, Canadian tobacco manufacturers exported tax-free Canadian brand cigarettes into the United States and then smuggled them back into Canada. The industry then used the evidence of smuggling (without admitting their role) to lobby the Canadian government to lower taxes. The excess consumption from the reduced excise taxes and directly smuggled (and cheaper) cigarettes were approximately 30–40 billion sticks in the early 1990s, leading to an estimated 30 000 to 40 000 tobacco-attributed deaths.^106 107^

Tobacco industry complicity in illicit trade was also the basis for large settlements with the European Union in 2004, 2007, and 2010. Philip Morris International, Japan Tobacco International, British American Tobacco, and Imperial Tobacco Limited agreed to pay more than $1 billion as part of agreements aimed at halting practices that supported smuggling.^108 109^ Additional cases have been documented in Asia, Eastern Europe, former Soviet republics, Latin America, the United States, and the United Kingdom.^108–110^

Third, smugglers increase illicit cigarette prices in line with increases in legal cigarette prices.^95^ Thus, even when increased taxes divert some demand to the illicit market, they push prices up in the illicit market as well, discouraging consumption.^96^ Recent evidence shows that illicit cigarette prices generally follow the prices of legal cigarettes (correlation coefficient: 0.87).^97 111^

For alcohol, there is less evidence of a relationship between taxation and illicit, unrecorded alcohol consumption. Unlike tobacco, alcohol is less susceptible to smuggling because it is heavier and more difficult to transport relative to its value. On the other hand, opportunities for artisanal, informal production are more widespread.^110^ WHO estimates show that the global share of unrecorded alcohol consumption fell from 28.6% in 2005 to 25.5% in 2016.^112 113^ Estimates of such a share for low- and lower-middle-income countries is around 43% for 2016, while for upper-middle and high-income countries, it is about 17.5%.^113^ Large variations also exist by region. The same can be said for SSB illicit trade, where there is a lack of any evidence relating higher SSB taxes to increased SSB illicit trade.^75^ The low price relative to volume for SSB is probably, a “natural” hurdle for illicit trading.

Increases in small-scale purchases of untaxed products (eg, bootlegging, cross-border shopping, etc.) have also been blamed as consequences of higher taxes and as reasons behind the failure of taxes to curb consumption. Evidence on sub-national SSB taxes for some US counties shows that, for instance, cross-border shopping exists but is not enough to offset the decreases in consumption that taxes produce.^92 114 115^ There is also evidence that the effect on aggregate consumption is small for alcohol and tobacco and it fades with distance from the border.^40 116^

### Taxes and economic activity and employment

According to the tobacco, alcohol, and SSB industries, excise taxes on these products reduce economic activity and employment when people purchase less of them. This argument, which contradicts their claims that taxes do not have an impact on consumption,^117^ is also simplistic and untrue. When taxes reduce the consumption of these products, it can affect sales and employment in those sectors. However, consumer spending on other products will increase and raise sales and employment in those other sectors.^118^ Furthermore, when governments spend the excise tax revenues, they also generate employment. Studies have found that shifting demand from the tobacco industry which is relatively capital-intensive to industries that are more labour-intensive can actually increase employment.^24^

A relatively large body of evidence exists on this for SSB taxes.^119^ Although the SSB industry fought against a tax increase in Mexico in 2014 by claiming it would reduce employment, subsequent evidence showed that the policy did not have any impact in terms of employment in the manufacturing sectors affected by the tax.^120^ Employment in the retail sector was also unaffected (it even showed a moderate increase). The same is shown for several cities in the US that implemented such a tax. In the case of San Francisco, a recent study showed that 2 years after the implementation of the SSB tax, there was no discernible effect on employment for the overall economy, private sector, supermarkets and other grocery stores, convenience stores, limited-service restaurants, and beverage manufacturing, when compared with a suitable synthetic group.^121^ Similar results have been obtained in studies simulating the impact of an SSB tax in California and Illinois.^122^ A study using synthetic control analyses showed that in the case of Philadelphia, trends in employment in key industries and in net total employment in the post-SSB tax period are not significantly different from trends in the pre-tax period.^92^

In the case of alcohol, a study simulated the effect of an alcohol tax increase on employment in six states and found that such a tax would have a positive impact on employment, mostly because the resulting fiscal expenditures would spur greater economic activity.^123^

Finally, in the case of tobacco, there is ample evidence that tax and non-tax policies (such as smoke-free policies) do not have a discernible effect on aggregate employment. Studies on this topic have been conducted for Scotland, the UK, the US, and some of its States (Michigan, Indiana), Canada, South Africa, Zimbabwe, Bangladesh, Bulgaria, Egypt, and Indonesia.^40^ In most cases, studies found a net gain of jobs under normal circumstances after a tobacco tax increase.

Though aggregate employment is not affected by tobacco taxes, there may be specific groups (eg, small-scale tobacco farmers) who may lose income or even go out of business. In most cases, farmers have long-established practices of shifting among crops in response to demand and market prices.^24^ In other cases, crop substitution programmes can be implemented. Alternatives to tobacco farming are present in countries as diverse as Indonesia, Malawi, Kenya, Brazil, Canada^40^ and China.^124^ Compensatory programmes can also be financed with tax revenues. For example, the Philippines earmarked a significant proportion (15%) of incremental revenues from tobacco taxes to help tobacco farmers shift to other crops.^125^ The benefits of reducing illnesses and deaths associated with tobacco consumption, and environmental costs from growing it, are substantially higher than the costs of implementing these crop substitution programmes.^126 127^

Non-tax measures, such as smoke-free policies, were also mentioned as hurting businesses (eg, bars, restaurants and pubs). However, studies conducted in several countries have shown little effect on sales, employment, number of establishments, business value, or gaming revenue.^40 128^

## Conclusions

In 1776, Adam Smith stated that “sugar, rum, and tobacco are commodities which are nowhere necessaries of life, which have become objects of almost universal consumption, and which are therefore extremely proper subjects of taxation.”^129^ Much time has passed since then and evidence on the negative effects of the consumption of tobacco, alcohol, and SSB is overwhelming (something unknown when Smith made that statement). In addition, economic theory has demonstrated that taxing products that generate negative externalities not only increases revenues but also increases economic efficiency.

Evidence collected over the past decades also shows that tobacco taxation is the single most effective intervention to curb tobacco use.^1 3 40 42^ Recently, WHO has included tobacco taxes as a “best buy” (interventions with the highest cost-effectiveness) to reduce consumption and the burden of diseases associated with its use.^130^ The same can be said for alcohol taxation. In the case of SSB taxes, although they have not been included in the “best buy” list to reduce unhealthy diets, they have been singled out as a cost-effective intervention for reducing SSB consumption and the most cost-effective to reduce SSB consumption.^130^

Reducing consumption of alcohol, tobacco, and SSB and the burden of disease associated with them is not only about reducing healthcare costs, which can be significant and put great pressure on health systems. It is also about increasing the social return on human capital. Chronic illnesses and conditions associated with the consumption of these products hinder individuals’ productive performance (due to absenteeism and presentism – reduction of productivity at work due to illnesses). Premature mortality (in the case of tobacco, for instance, smokers lose on average a decade of life when compared with non-smokers)^4^ implies that social resources devoted to, for instance, education and health, are not fully realised and are prematurely lost. Loss of income due to illness and mortality may affect households' present and future well-being, creating a vicious cycle of lower human capital investment and poverty.

Contrary to the simplistic arguments made by affected industries, higher taxes on these harmful products are highly progressive (ie, pro-poor). Because poorer individuals are more price-responsive, they have a higher propensity to reduce consumption or quit altogether. Consequently, they benefit disproportionately from longer healthier lives, reduced spending on healthcare, fewer lost days of work, and longer working lives.^76 131^ Evidence on this for countries of different income levels is significant and consistent.

Unfortunately, in too many countries the use of these taxes is still far from optimal. Most governments have only enacted modest tax increases instead of the kind of large excise hikes paired with non-price strategies which will be most effective at reducing consumption. A global assessment of tobacco taxes documented significant progress but noted that it has been slow and remains inadequate.^132^

Taxes are an important element of broader efforts to reduce consumption of tobacco, alcohol, and SSB. They are not a cure-all. They should be used along with other cost-effective measures. These measures include mass media education campaigns, bans on smoking or drinking in public places, prominent labelling showing adverse health effects (especially for tobacco and alcohol); restrictions on opening times (for alcohol); restrictions on smoking and drinking alcoholic beverages in public spaces; labelling of products with health warnings (for tobacco, alcohol, and SSB); etc.^130^ There is enough evidence on the effectiveness of these measures to amply justify their implementation, with adaptation and prioritisation depending on individual country circumstances.

## Data Availability

No data are available.
